# Case Management of Severe Malaria - A Forgotten Practice: Experiences from Health Facilities in Uganda

**DOI:** 10.1371/journal.pone.0017053

**Published:** 2011-03-01

**Authors:** Jane Achan, James Tibenderana, Daniel Kyabayinze, Henry Mawejje, Rukaaka Mugizi, Betty Mpeka, Ambrose Talisuna, Umberto D'Alessandro

**Affiliations:** 1 Department of Paediatrics and Child Health, Makerere University College of Health Sciences, Kampala, Uganda; 2 Malaria Consortium Africa, Kampala, Uganda; 3 London School of Hygiene and Tropical Medicine, London, United Kingdom; 4 Communicable Disease Research Programme Consortium, Malaria Consortium, Kampala, Uganda; 5 Department of Epidemiology and Biostatistics, Makerere University School of Public Health, Kampala, Uganda; 6 Department of Parasitological, Institute of Tropical Medicine, Antwerp, Belgium; Université Pierre et Marie Curie, France

## Abstract

**Introduction:**

Severe malaria is a life-threatening medical emergency and requires prompt and effective treatment to prevent death. There is paucity of published information on current practices of severe malaria case management in sub-Saharan Africa; we evaluated the management practices for severe malaria in Ugandan health facilities

**Methods and Findings:**

We did a cross sectional survey, using multi-stage sampling methods, of health facilities in 11 districts in the eastern and mid-western parts of Uganda. The study instruments were adapted from the WHO hospital care assessment tools. Between June and August 2009, 105 health facilities were surveyed and 181 health workers and 868 patients/caretakers interviewed. None of the inpatient facilities had all seven components of a basic care package for the management of severe malaria consistently available during the 3 months prior to the survey. Referral practices were appropriate for <10% (18/196) of the patients. Prompt care at any health facility was reported by 29% (247/868) of patients. Severe malaria was correctly diagnosed in 27% of patients (233).Though the quinine dose and regimen was correct in the majority (611/868, 70.4%) of patients, it was administered in the correct volumes of 5% dextrose in only 18% (147/815). Most patients (80.1%) had several doses of quinine administered in one single 500 ml bottle of 5% dextrose. Medications were purchased by 385 (44%) patients and medical supplies by 478 patients (70.6%).

**Conclusions:**

Management of severe malaria in Ugandan health facilities was sub-optimal. These findings highlight the challenges of correctly managing severe malaria in resource limited settings. Priority areas for improvement include triage and emergency care, referral practises, quality of diagnosis and treatment, availability of medicines and supplies, training and support supervision.

## Introduction

Severe malaria is a life threatening medical emergency that requires prompt and effective treatment to prevent death.[1], [2] However, effective management of severe malaria is relatively expensive and relies heavily on well equipped hospitals, with adequately trained health workers, both often lacking in sub-Saharan Africa.[3], [4] Severe malaria has been described as a neglected disease that poses a significant economic burden on most African countries which typically have weak health systems and are unable to finance basic services and infrastructure. [5]

In Uganda, efforts to improve the management of severe malaria at formal health facilities started in 1998, [6] mainly through training workshops using adapted WHO training materials. Despite these efforts, severe malaria management remains challenging, as it depends on the availability of treatments, blood transfusion services, functional referral systems, good infrastructure and adequate organization of hospital services. There is limited information on management practices for severe malaria in resource constrained settings in Africa, with few studies reporting on this as part of integrated pediatric care evaluations.[7], [8] We evaluated these practices at different levels of health care in Uganda.

## Methods

### Ethics statement

The study was approved by the Uganda National Council for Science and Technology and verbal consent was obtained from all participants. Verbal consent was considered more appropriate than written consent for this survey as this was considered a routine audit/evaluation of health services. Verbal consent was documented as a tick on each case record form.

### Study design and setting

Between June and August 2009, a cross sectional assessment of severe malaria management practices was conducted in selected health facilities in 11 districts in Uganda. For patients with severe malaria, hospitals and health centre IVs run by specialists, medical officers and clinical officers provide inpatient services while health centre IIs and IIIs run by nurses typically provide outpatient and referral services.

### Sampling methodology

Multi-stage sampling methods were used to select study sites. The eastern and mid-western regions of Uganda were selected to represent areas of high and low - medium malaria transmission settings, respectively. Out of 15 districts in these regions, 11 were randomly selected; 6 in eastern Uganda (Kumi, Soroti, Katakwi, Bukedea, Amuria and Kaberamaido) and 5 in mid-western Uganda (Bulisa, Hoima, Kibaale, Kiboga and Masindi). Within the districts, in order to obtain a representative sample of health facilities for each region, all hospitals and health centre IVs (in-patient facilities) were selected while among the 250 health centres II and III (lower level facilities) 30% were randomly selected. In all the selected health facilities, the director and the health workers involved in any aspect of care of malaria patients and available during the survey days were interviewed. In addition, after having obtained their or caregivers' verbal consent, randomly selected patients having malaria according to the admission register and hospitalized during the survey days were interviewed.

### Data collection

The survey was conducted by 5 teams of 4 to 8 health workers working in parallel. The teams were trained for 1 week prior to the survey to ensure that interview questions were appropriately asked and responses consistently recorded. Training and concordance testing was done until the agreement of practice results of interviewers and trainers was >90%. District officials and health unit directors were informed about the survey only on the morning of the survey. A triangulation approach was used to collect data with the following methods: health facility assessments and health worker interviews at inpatient and lower level facilities as well as in-patient/caregiver interviews and reviews of patient's charts at inpatient facilities. Most survey instruments were adapted from the WHO hospital care assessment tools. Survey instruments can be found at www.plosone.org (Appendices S1, S2, S3, S4, S5, S6). Study coordinators reviewed all survey tools daily for completeness and accuracy. Health facility assessments collected information on staffing, triage systems, emergency care, presence of malaria treatment guidelines, laboratory practices and availability of medicines and supplies. Health worker assessments collected information on knowledge of severe malaria and its management, prescribing practices, training and support supervision. Knowledge on severe malaria management was further assessed using a clinical case scenario of a patient presenting with fever, convulsions and loss of consciousness. In-patient/caregiver interviews and chart reviews collected information on presenting complaints, time taken to receive care, diagnosis, patients' weight, laboratory investigations and treatment prescribed. On average, 17 patients were recruited in each health centre IV and 66 patients in each hospital. Patients/caregivers were asked to report their satisfaction with services provided on an ordinal scale (good, improvement needed or poor) and to suggest improvements. Any information not obtained through these two approaches was considered not documented. For missing weights we used a weight equivalent to the 50% percentile for age according to the 2000 CDC growth charts. [9]

### Definitions

Severe malaria case management was assessed according to the following definitions: correct diagnosis: documented fever or history of fever with a positive malaria test and at least one sign/symptom of severe disease according to WHO criteria[10]; prompt management: patient with severe malaria receiving care within 30 minutes of presentation at the health facility; correct initial parenteral antimalarial medicine prescribed: administration of parenteral quinine, artemether or artesunate; correct antimalarial drug dose and dosing regimen: IV quinine10 mg/kg every 8 hrs (margin of error +/−20 mg on total daily dose) or IM artemether 3.2 mg/kg on day 1, followed by 1.6 mg/kg daily or IV artesunate 2.4 mg/kg on admission at 12 hrs and then every 24 hrs (margin of error +/−5 mg on total daily dose); all given until the patient was able to tolerate oral therapy. [10], [11] Correct mode of administration: IV quinine in 10–20 ml/kg of 5% dextrose, intramuscular administration of artemether or IV artesunate mixed with 5 mL of 5% dextrose and injected as a bolus; appropriate oral continuation therapy after initial parenteral treatment: either oral quinine at 10 mg/kg every 8 hrs until completion of a 7-day course or a full treatment course of an oral artemisinin based combination therapy according to appropriate weight-based dosing guidelines; [10] adequate referral practice: referral of a patient with severe malaria after administration of injectable quinine or rectal artesunate, provision of a referral note and transport[10], [11]. Patients were considered appropriately treated if they received the correct antimalarial medicine, at the right dose and dosing regimen and with the correct mode of administration.

### Sample size estimation, data management and analysis

For the inpatient interviews, a sample size of 869 inpatients was estimated assuming 50% of malaria inpatients are appropriately treated, at 95% level of confidence, with a tolerable error of 0.05, a design effect of 2 and allowing for 10% non-responsiveness.

Data were double entered in EPI-info software program version 6 and analysed using STATA version 10.0 (StataCorp LP, College Station, TX, USA). Results from all districts were combined and descriptive analysis was done at health facility, health worker and patient levels. Data are presented as proportions and frequencies adjusted for clustering by health facility. Fisher's exact tests were used to analyze differences in proportions. Two tailed p values and a 5% significance level were used.

## Results

In the 11 districts, 105 health facilities were included (83 lower level facilities and 22 inpatient facilities) and 181 health workers interviewed (151 at lower level and 50 inpatient facilities respectively). In addition, 868 inpatient interviews and chart reviews were conducted. No health worker or caregiver declined to participate.

### Health facility characteristics

The majority of health facilities (83%, 87/105) were government-run institutions. Despite health workers' reports of a defined triage system in most health facilities, triage was practised in less than half (44%, 46/105) of them (Table 1). Only 11.4% (12/105) of health units had separate outpatient (OPD) queues for adults and children. Functional microscopes for malaria diagnosis were available in most inpatient units (77.3%, 17/22) and in about half (51.4%, 18/35) of the health centre IIIs (Table 1). Malaria rapid diagnostic tests were available in 14.4% (12/83) of health centre IIs and IIIs. Haemoglobin measurement was available at 39% (41/105) of the facilities.

**Table 1:** Health facility characteristics.

Characteristics	N = 105	
	No.	%
**Health facility level**		
Health centre II	48	(45.7)
Health centre III	35	(33.3)
Health centre IV	12	(11.5)
District hospital	8	(7.6)
Regional Referral hospital	2	(1.9)
**Type of Health facility**		
Government	87	(82.9)
Faith based	14	(13.3)
Private for profit	4	(3.8)
Treatment aide memoirs in outpatient units available	83	(79.0)
Health facilities with defined triage system	82	(78.1)
Triage practised	46	(43.8)
Presence of separate lines for adults and children in OPD	12	(11.4)
Functional weighing scale available	79	(75.2)
Thermometers available	83	(79.0)
**Antimalarial medicines available on the day of survey**		
Quinine injection	79	(75.2)
IV artesunate	2	(1.9)
Rectal artemisinin	5	(4.8)
Artemether Injection	10	(9.5)
Quinine tablets	41	(39.0)
Artemether-lumefantrine tablets	52	(49.5)
Sulphadoxine-pyrimethamine tablets	64	(60.9)
‡ **Antimalarial medicines available in the 3 months prior to survey**		
Quinine injection	57	(54.3)
Quinine tablets	17	(16.2)
Artemether-lumefantrine tablets	35	(33.3)
‡,† **Supplies for severe malaria management available in the 3 months prior to survey at the inpatient units (N = 22)**		
5% dextrose	8	(36.4)
50% dextrose	7	(31.8)
Blood for transfusion	1	(4.5)
Blood transfusion sets	8	(36.4)
IV giving sets	10	(45.5)
Availability of seven basic medicines and supplies for severe malaria management in the 3 months prior to survey at inpatient units*	0	(0)
† **Malaria testing facilities available**		
Functional microscope at inpatient units (N = 22)	17	(77.3)
Functional microscope at health centre IIIs (N = 35)	18	(51.4)
RDTs at health centre IIs and IIIs (N = 83)	12	(14.4)

*Basic package includes: Quinine injection, Intravenous fluids, 50% dextrose, blood for transfusion, IV giving sets, blood transfusion set, Syringes.

‡ Stock-outs defined as the absence of medicines or supplies for >1 week in the 3 months prior to the survey.

† Denominators indicated in brackets.

During the 3 months prior to the survey, 54.3% (57/105) of health facilities had consistent availability of parenteral quinine, while fewer facilities had quinine tablets (16.2%, 17/105) and artemether-lumefantrine tablets (33.3%, 35/105). None of the inpatient facilities had consistent availability of all seven components of a basic care package for severe malaria management (parenteral quinine, intravenous fluids, 50% dextrose, blood for transfusion, transfusion sets, IV giving sets, syringes). The most common stock outs were blood for transfusion (available in 4.5% of units), 50% dextrose (in 32%), 5% dextrose and transfusion sets (in 36.4%) (Table 1).

### Health worker characteristics

At the inpatient units, nurses/midwives represented the majority of the staff (40%). Considering all health facilities visited, only 2 doctors were on duty on survey days. The percentage of health workers who could mention >2 severe forms of malaria was 24% at the inpatient units and only 2.3% at the lower levels of care. In response to the clinical case scenario, 52% (26) of health workers at the inpatient level and 49.6% (65) at the lower levels of care were able to write an accurate prescription for a 4 year old patient (Table 2). Regarding on-site training, 22.2% (28/131) of health workers at the lower levels of care and 22.0% (11/50) at the inpatient units reported having received in-service training on severe malaria management within the year prior to the survey. Fewer health workers at the inpatient units (24.0%, 12/50) than those at the lower levels of care (41.9%, 55/131) (p = 0.025) reported having received at least one support supervision visit in the previous 6 months. (Table 2)

**Table 2:** Health worker Characteristics.

Characteristics	Lower level units: Health centre II and III (N = 131) No. (%)	Inpatient units: Hospitals and Health centre IV (N = 50) No. (%)	P value
**Pre-service training**			
Medical officer	0	2 (4.0%)	0.02
Clinical officer	9 (6.9%)	14 (28.0%)	0.00
Nurse/midwife	36 (27.5%)	20 (40.0%)	0.12
Nursing aide/assistant	86 (65.7%)	14 (28.0%)	0.00
In service at current post for >12 months	97 (74%)	39 (78.0%)	0.58
Diagnosis of malaria based on clinical features and diagnostic tests (confirmatory)	11 (8.9%)	26 (52.0%)	0.00
Health worker ever undergone IMCI training	62 (49.6%)	28 (56.0%)	0.47
Received in-service training on severe malaria case management in last 12 months	28 (22.2%)	11 (22.0%)	-
Health worker has malaria treatment guidelines accessible	108 (82.4%)	43 (86.0%)	0.52
**Knowledge on severe malaria**			
**Common forms of severe malaria listed**			
Severe anaemia	6 (4.6%)	38 (76%)	0.00
Repeated convulsions	8 (6.1%)	35 (70%)	0.00
Cerebral malaria	93 (71.0%)	27 (54%)	0.03
Hypoglycaemia	14 (10.7%)	17 (34%)	0.00
Shock	2 (1.5%)	15 (30%)	0.00
Spontaneous bleeding	65 (49.6%)	5 (10%)	0.00
Pulmonary oedema	18 (13.7%)	1 (2)	0.02
**Response to hypothetical clinical case**			
Correct antimalarial medicine choice	113 (89.7%)	48 (98%)	0.03
Correct quinine prescription for child	65 (49.6%)	26 (52%)	0.81
Correct quinine prescription for adult	93 (71.1%)	40 (80%)	0.22

### Patient assessment and emergency care

The majority of patients (76.3%, 663/868) were aged <5 years; the median age being 2 years. Fever or history of fever was the commonest reason for attendance (96.6%). Mean duration of hospitalisation at the time of interview was 2.5 days (SD 1.5), with 546 patients (62.9%) hospitalised for ≤2 days, 221 (25.5%) for ≤1 day and 142 (16.4%) for ≥4days. Malaria or severe malaria was the diagnosis documented in 93.8% of patients (814/868). Among these, 103 (11.9%) were recorded as malaria with severe anaemia (45% confirmed by microscopy) and 21 (2.4%) as cerebral malaria (57% confirmed by microscopy).

The median waiting time before receiving care at the facility was 3.0 hours (range 0–24 hours) with 28.5% (247/868) of patients reporting having received care within the first 30 minutes and 52.3% (454/868) within 1 hour of attendance. At least 33 patients (3.8%) waited ≥8 hours before receiving any care. Though most patients were asked about their age (96.3%), history of fever (89%), prior use of antimalarial therapy (58%) and history of repeated vomiting (55%), patients/caretaker reports and chart reviews revealed that presence of common danger signs were not often elicited (history of convulsions in 303 (35%) and drowsiness in 248 (29%) patients). Body temperature and level of consciousness were assessed in 20.5% (178/868) and 23.6% (205/868) of patients, respectively. The proportion of patients with at least one sign or symptom of severe malaria documented was 27.9% (242/868). Malaria infection was confirmed by microscopy in 64.7% (432/668) of patients in health facilities where functional microscopy was available.

### Case management practises

One hundred ninety six patients (23%) had been referred from a lower level of health care. The main reasons for referral were: poor response to treatment (38%) or unavailability of either blood for transfusion (33%), intravenous fluids (18%) or beds (9%). Pre-referral medications were given to 145 (79%) patients, quinine in 39.3% (57/145), often administered alone (65%, 37/57), or with an antibiotic (13%), an antipyretic (15%) or diazepam (5%). No patient received pre-referral rectal artesunate, referral notes were provided for 58.7% (115/196) and transport for only 6.1% (12/196). Overall, 9.2% (18/196) patients referred had adequate referral practises; 1.3% (1/76) in the low-medium transmission setting and 14.2% (17/120) in the high transmission setting (p = 0.002).

Two hundred thirty three (27%) patients had a correct diagnosis of severe malaria. The proportion of patients with a correct diagnosis was higher in the high transmission setting, 29.7% compared to 17.9% in the low-medium transmission setting (p = 0.001). Most patients were evaluated at least once a day during their hospitalization, though 102 (13%) were never assessed (Table 3). The majority of patients (95%, 823/868) received the correct initial parenteral antimalarial medicine, often at the recommended dose and dosing regimen (70.4%, 611/868). However, the dose was inappropriate in all 8 patients treated with artemether. For patients treated with quinine, 75% (611/815) were correctly dosed, 12.7% (104/815) were under dosed and 12.3% (12.3%) over dosed. Among patients treated with quinine, 18% (147/815) received the correct dosing regimen and mode of administration. Significantly, in most cases (75%) multiple doses of quinine were administered in a single 500 ml bottle of 5% dextrose to run over 24 to 48 hours. The proportion of in-patients with a negative blood smear but receiving antimalarial treatment was 94.9% (129/136). Overall, only 16.9% of the patients were appropriately treated for severe malaria. (Table 3) Medications needed for treatment were purchased by 385 (44%) and medical supplies by 478 patients (70.6%) at a mean cost of $2.8 (SD 2.9) and $3.4 (SD 3.7), respectively.

**Table 3:** Case management practises for patients hospitalised with a diagnosis of malaria.

	N = 868		
	No.	%	95% CI(Cluster adjusted)
Patients with a negative blood smear receiving antimalarial treatment (N = 136)	129	94.9%	87.8–100
**Reported frequency of evaluation by health workers during hospitalization**			
Once every day	410	50.1	
Twice or thrice daily	265	32.4	
Never seen	102	12.5	
**Patients purchasing medications**	385	44	34.8–53.9
Purchased medications	214	44.5	
Quinine	76	15.8	
Antibiotics	38	7.9	
Haematinics			
**Patients purchasing medical supplies**	478	70.6	61.7–79.5
Purchased medical supplies	223	33.4	
Intravenous cannula	162	24.3	
Intravenous fluids	109	16.3	
Syringes	101	15.0	
Giving sets	27	4.0	
Gloves			
**Correct antimalarial treatment**	823	94.8	91.7–98.7
**Initial parenteral antimalarial medicine prescribed**			
Quinine	815	93.9	
Artemether	8	0.9	51.9–87.3
Initial parenteral antimalarial medicine dose and dosing regimen	611	70.4	12.0–21.9
Initial parenteral antimalarial medicine, dosing regimen and mode of administration (appropriately treated)	147	16.9	
**Oral continuation therapy (n = 486)**	429	88.3	
Oral quinine	274	63.9	28.8–85.3
Artemether-lumefantrine	149	34.7	15.1–47.0
Dihydroartemisinin-piperaquine	6	1.4	0–3.4

Almost half of the patients (43.3%) considered that they had waited too long before seeing any health worker at presentation and 45% thought that services offered needed further improvements. Quality of care at the health facilities was reported as good by 46.8% of patients/caretakers, 45% thought that services offered needed to be improved while 8.2% thought services were poor. Suggestions for improvement included having sufficient medicines at health units (21.3%), improving the availability of supplies and sundries (11.6%), increasing the number of staff (8.3%), providing more beds and beddings (7.6%) and health workers having better attitudes towards patients and attendants (7.1%).

## Discussion

In our survey, management of severe malaria in Ugandan health facilities was sub-optimal, with most facilities not fully complying with the national and international treatment guidelines. We found significant problems with case management at both the health system/health centre and provider levels. Indeed, this survey identified several problems at different levels of the health care system, from the referral practices at the lower level health centres to the availability of supplies and actual management of malaria cases in referral facilities. Despite the existence of some differences between the two regions, the problems identified in the management of severe malaria cases were similar, indicating that both regions need similar attention and efforts to improve this unacceptable situation. Though the quality of documentation may have impacted on our assessment, we believe these findings accurately represent the management practices in these settings.

Practices related to severe malaria case management were deficient, from patient evaluation, for which the presence of danger signs were not systematically checked, to diagnosis, correctly done in <30% of patients, and treatment, which was usually correct in terms of dose and dosing regimen but for which drug administration was often not done as recommended. Deficiencies in correctly diagnosing severe malaria suggest that a significant proportion of these patients may have had uncomplicated malaria and did not require parenteral therapy or hospitalisation. This calls for measures to improve patient evaluation and promotion of the rational use of antimalarial medicines. Furthermore, none of the inpatient health facilities had all components of a basic care package for severe malaria management available, with blood for transfusion, 5% dextrose, and transfusion sets least available.

Though the survey was not designed to evaluate the impact of management practices on clinical outcome, it would be expected that such shortcomings would influence patient survival. The large majority of patients included in this survey had already gone through the first 24–48 hours of hospitalization, a known critical period,[12] and may not fully represent treatment practices in those with a fatal outcome. Therefore, the quality of case management might be worse than documented here.

When analysing these observations in more detail, patient triage, evaluation and diagnosis were extremely inadequate. More than half of health facilities did not practise triage and few had separate OPD queues for adults and children, an important element as the large majority of the patients were children <5 years of age. Such inefficient systems may explain the long waiting times prior to receiving care at the health facilities. Good quality emergency care and triage is a critical first step in improving hospital care; unfortunately, triage is often deficient in resource limited settings. [7], [13]This worrying finding can be addressed by training health workers on emergency triage, assessment, and treatment [14] and by providing practical support through supervision and clinical audits. This strategy would not only improve the management of severe malaria cases but also that of other severely ill patients. Though health worker training has been shown to be critical for improving case management, [15]–[16]the cadre of health workers to be targeted needs to be critically reconsidered. In our setting, nurses and nursing aides, though not primarily responsible for clinical management decision taking, should have the priority as they were the only cadre of staff consistently available at the units whereas medical and clinical officers, who theoretically have the primary responsibility, were consistently absent.

The proportion of in-patients with a negative blood smear but receiving antimalarial treatment was substantial. This finding has previously been reported in similar settings, with an increased risk of death in these patients when treated for malaria, possibly due to inappropriate treatment of other illnesses [17], [18], [19]. In our setting, there was also significant concurrent administration of antibiotics that could be attributed to diagnostic uncertainty. Routine treatment with parenteral antibiotics may be warranted, particularly when microscopy is not available or of insufficient quality, because of the increased risk of bacterial sepsis and associated mortality in malaria patients [20]. The recent decision by the Ugandan Ministry of Health to have all suspected malaria cases confirmed by microscopy or rapid diagnostic test may improve diagnosis. The challenge though remains to ensure consistent availability of these tools at all facility-based service delivery points.

The adequacy of treatment dose, dosing schedule and oral continuation therapy in our survey is reassuring; the latter was probably due to recent in-service training conducted on the management of uncomplicated malaria at the time of treatment policy change in Uganda in 2006. However, the method of quinine administration is a cause of concern. Most patients had multiple doses of quinine (for 24 to 48 hours) combined in a single 500 ml bottle of 5% dextrose. The rationale for this practise is unclear; but may be due to the desire to minimize costs. Nevertheless, this practice is concerning and should be discouraged as it increases the risk of both quinine toxicity and fluid overload, particularly in children. The provision of smaller volume bottles for infusion, more suitable for paediatric patients, may overcome this problem. In addition, the use of artesunate injections may further improve treatment delivery as this regimen does not require rate-controlled infusion. The SEAQUAMAT[21] and recently published AQUAMAT study [22] provide sufficient evidence of the superiority of artesunate over quinine in both children and adults and this should lead to severe malaria treatment policy change to intravenous artesunate in several Sub-Saharan countries, including Uganda. In our study, the alternative to quinine in a few patients was artemether, which was always administered at an incorrect dosage, possibly because the heath providers had little experience with this product.

Importantly, stock-outs of several items included in the basic care package for severe malaria management were common and could explain the high proportion of patients obliged to purchase medications and supplies needed for their management. This is certainly a major challenge, as improving clinical skills through training without ensuring availability of medicines and supplies will have limited impact on the quality of care. These shortages impact negatively on efforts to deliver effective treatment and undermine malaria control efforts. [23] Such stock-outs are caused by different factors and often reflect weaknesses in medicine and supplies procurement, management and distribution practices. Indeed, in this study inadequate and delayed funding, delayed drug deliveries and poor storage were identified as the main causes of stock-outs (data not presented). This problem must be addressed by the Ugandan Ministry of Health as a matter of priority to improve the quality of care and minimize out-of-pocket costs incurred by patients/caregivers, which were unacceptably high for a country like Uganda where 52% of the population lives below the international poverty line of US$1.25 per day[24]. Shortages may also be curtailed by measures to improve severe malaria diagnosis and by the use of diagnostic tests to improve targeting of treatment. It is also critical for resource limited countries like Uganda to look for more efficient ways of financing health care as the current system does not seem to mobilize sufficient resources to provide the desired levels for the entire population [25]. At the national and international level, much more attention appears to be focused on community-based health care interventions. There is need to shift some of this attention back to facility-based health care services, especially since community service delivery is linked to that at health units.

In conclusion, this study highlights the serious challenges faced in the management of severe malaria in a resource limited setting like Uganda. There is paucity of published information on current severe malaria management practices in sub-Saharan Africa, but the situation in many areas may not be very different from what we have observed in Uganda. Considering the problems identified, several priority areas at different points of care needing improvement would include: patient assessment, referral practices, quality of diagnosis, triage and emergency care, treatment practices, availability of medicines and supplies, health worker training and support supervision. Considering its huge toll on African children, improved management of severe malaria should be a priority.

## Supporting Information

Appendix S1 Severe malaria survey tool for Inpatient Interview.
(DOCX)

Appendix S2 Severe malaria survey tool for Outpatients' Health Centre II and III.
(DOCX)

Appendix S3 Severe malaria survey tool for Inpatient unit/ward.
(DOCX)

Appendix S4 Severe malaria survey tool - Checklist for each Health Facility.
(DOCX)

Appendix S5 Severe malaria survey tool for pharmacy.
(DOCX)

Appendix S6 Severe malaria survey tool for laboratory.
(DOCX)
