# Supplementary material for: Case Management of Severe Malaria - A Forgotten Practice: Experiences from Health Facilities in Uganda
Source: PLoS One. 2011 Mar 1;6(3):e17053. doi: 10.1371/journal.pone.0017053 (PMC3046961; doi:10.1371/journal.pone.0017053)
Supplement: Appendix S1 — Severe malaria survey tool for Inpatient Interview. (DOCX) [file pone.0017053.s001.docx]

# Appendix S1: Severe malaria survey tool for Inpatient Interview

Date__/___/___

***Instructions***

*1. Complete the blank space with the answers given*

*2. Select the most appropriate option by clearly ticking the correct one/s with a pencil.*

*3. Do not prompt with the listed answers unless prompting is specified*

*4. If the Health centre III does not have admission facilities then use the form for outpatients*

**A. Geographic and Demographic information (GD)**

1. Name of Health facility:_____________________2. Status (circle one): HCII, HCIII, Hospital

3. Facility code (circle one): H (High malaria transmission) L (Low malaria transmission)

4. Patient’s names Initial_____________________ 5. IP number_____________________

6. Patient’s age: __________________________7. Sex (circle one): M (Male), F (Female)

8. Caretaker characteristics (circle one): M (Male), F (Female)

9 Caretaker relationship to patient: (circle one)

1. Biological mother
2. Biological father
3. Spouse
4. Other relative (Specify)___________________


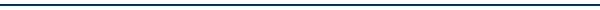


**B. Medical History (MH)**

1. Why did you come to the health facility (complaints at admission)
   1. Fever or h/o fever (Y/N)
   2. Diarrhoea/vomiting (Y/N)
   3. Fast breathing/ difficulty in breathing (Y/N)
   4. Child difficult to wake (Y/N)
   5. Child convulsing (Y/N)
   6. Ear problem (Y/N)
   7. Other (Specify)__________________________________

2 How long did the patient wait before receiving medical care?............................(.hours)?

3 Was your patient immediately assessed and given priority treatment over others? (triage)…………(Y/N)

1. For how long have you/your patient been on the ward?______-(days)

Ask the patient or caretaker if at the time of admission they were asked the following questions:{Y/N}

1. .Does the patient’s present illnesses involve a fever? [ ]
2. If yes, was fever present in last 24 hours? …………………… [ ]
3. Did patient receive any antimalarial/antibiotic for this illness prior to this visit [ ]
4. Was the patient asked which name and formulation of the medicine taken [ ]
5. Did the patient have convulsions? [ ]
6. Did the patient become drowsy, confused or lose consciousness? [ ]
7. Was the patient extremely weak, unable to sit or stand……………………………. [ ]
8. Was the patient unable to eat or drink or refused to breastfeed? [ ]
9. Did the patient suffer from cough?.............................................................. [ ]
10. Did the patient suffer from breathing problems?................................................... [ ]
11. Did the patient vomit repeatedly?............................................ .[ ]
12. Did the patient have diarrhoea?........................................... [ ]
13. Did the patient have yellow eyes (Jaundice)?........................................................... [ ]
14. Did the patient suffer from stomach ache?.................................................................[ ]
15. Did the patient have any ear problems like discharge ......... [ ]
16. Does the patient suffer from any underlying illness(Y/N)………………………… .[ ]
17. Are you on any medication for that illness? (Y/N)……………………… [ ]
18. Does any of the patients contacts suffer from a similar condition?......................... [ ]


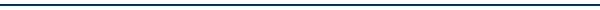


**C. Examination and Investigations (EI)**

1. Did any HW **ask/record patient’s age** during this visit? [__]
2. Did any HW **measure weight**? (Y/N) [__]
3. Did any HW **measure temperature**? (Y/N) [__]
4. Did any HW take the patient’s pulse (Y/N)… [__].
5. Did any HW take the patients pressure? [__]
6. Did the health worker physically examine the patient? (Y/N)………………………[__]
7. If yes, which of these procedures did he/she perform?

Examine (look at or touch) the following

- Eyes…………………….
- Ears……………………….
- Mouth…………………….
- Tongue…………………..…..
- Skin for lesions,
- Chest with a stethoscope……………….
- Take pulse (feel the wrists)…………………………….
- Touch the abdomen………………….
- Level of consciousness (Try to arouse patient)…………………………..

8. Was the patient sent to the laboratory for investigations (Y/N) [__]

What specimen (samples) were taken

- 1. blood
  2. blood smear ( on a glass slide)
  3. urine
  4. stool
  5. CSF ( water off the back)


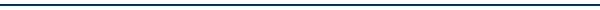


#### D. Communication and counseling

Answer Yes or No for all the questions below

1. Did the HW offer you or your patient reassurances? (Y/N) [ ]
2. Did they explain the diagnosis [ ]
3. Did the explain to you the treatment given (Y/N) [ ]
4. Did the HW tell you how often to take the medicine [ ]
5. Where you told who to call when the patients condition worsened [ ]
6. Did they seek consent for the tests? (Y/N) [ ]
7. Did they interpret results to the patient and care givers? (Y/N) [ ]
8. Did the HW tell you to continue feeding or breastfeeding? (Y/N) [ ]
9. Did the health worker educate you on any health issues like Immunization [ ]
10. Did the HW talk to you about referral? (Y/N) [ ]
11. Did the HW ask you to return immediately if you or your child **becomes sicker**? [ ]
12. Were you or your patient given any injectable antimalarials?
13. Yes
14. No
15. Doesn’t know

13. If yes:

- 1. What was the name of the injection?

___________________________________________________

- 1. What was the injection for?
  2. How was the injection given? [ ]
     1. Intravenous (In a drip) (Y/N)
     2. On the buttocks (Y/N)
     3. On the thighs (Y/N)
     4. Not sure (Y/N)
  3. How many injections were you given per day? [ ]
     1. One (Y/N)
     2. Two (Y/N)
     3. Three (Y/N)
     4. Other, Specify__________________________
  4. For how many days were the injections given?
     1. One (Y/N)
     2. Two (Y/N)
     3. Three (Y/N)
     4. Four (Y/N)
     5. Five (Y/N)
     6. Other, Specify_______________________

Did you buy any medicines that were not available in the hospital? [ ]

What medicine did you buy _____________________________

How much did you spend on these medicines?________________________

Did you buy any medical supplies [ ]

List what you bought _________________________________________

How much did this cost you?_____________________________________


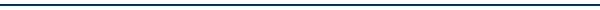


**C. Satisfaction with care given**

1. What do you think of the services provided at this facility? (read all options to the patient/caretaker)
   1. Good as they are
   2. Should be improved. If this option chosen, list what should be improved
      1. ____________________________________________________
      2. _____________________________________________________-
      3. ______________________________________________________
      4. ______________________________________________________
   3. Doesn’t know
2. What do you think about the time you had to wait to see the health worker on the day you/your patient was admitted? (read all options to the patient/caretakers)
   1. Definitely too long
   2. Long
   3. Acceptable
   4. Short
   5. Doesn’t know
3. While on the ward, how often were you/your patient examined by the health workers? (read all options to the patient/caretakers)
   1. Once every day
   2. Twice daily
   3. Three times daily
   4. On alternate days
   5. Once in three days
   6. Never seen
   7. Others, Specify_________________________

1. What type of medication did the health worker give or prescribe for you or your patient?
   1. Injectables
   2. Oral medication
   3. Both


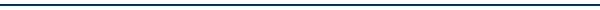


**E. Patient triage (PT)**

1. How long did the patient wait before receiving medical care?................................?

2Was your patient immediately assessed and given priority treatment over others?(triage)…………(Y/N)

3. Were there health workers look out for very sick patients and getting them quick attention (Y/N) [ ]


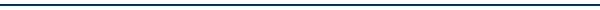


**C. Referral History**

1. Where you referred to this facility from a lower health centre? (Y/N) [ 2.What type of facility __________________________________

3. Where you given any pre-referral medication [ ]

4. What medicines were you given_____________________________

3. What were the reasons why you were referred?

i. Lack of blood for transfusion at the facility (Y / N)

ii. Poor response to treatment given (Y / N)

iii. Lack of I.V fluids (Y / N)

iv. Lack of Oxygen (Y / N)

v. No beds available to admit patient (Y / N)

vi. Others, specify___________________________________

4. Were you given any support to get to this health facility? (Y/N)

5. What support were you given to help you get to this health facility

____________________________


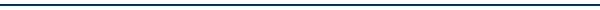


**F Record reviews for the patient: (R)**

**Medical record number: ____________________________________**

***Section 1: History***

| **Is the clinical history documented in the medical record?**  *Please indicate if the symptom is commented on in the medical record, not whether the symptom was present or absent.* | | | | |
| --- | --- | --- | --- | --- |
| **History / Symptom** | **Recorded?** | **History / Symptom** | | **Recorded?** |
| Age | □ Yes □ No | Loss of consciousness | | □ Yes □ No |
| LC1 chairman name | □ Yes □ No | Refusal to feed / breastfeed | | □ Yes □ No |
| Fever | □ Yes □ No | Vomiting | | □ Yes □ No |
| Weakness | □ Yes □ No | Diarrhea | | □ Yes □ No |
| Pallor | □ Yes □ No | Cough | | □ Yes □ No |
| Jaundice | □ Yes □ No | Fast breathing | | □ Yes □ No |
| Convulsions | □ Yes □ No | Edema | | □ Yes □ No |
| **Is the past history documented in the medical record?**  *Please indicate whether the following aspects of the history are commented on in the medical record.* | | | | |
| Comment on past medical history:  □ Yes □ No | | | Comment on gestational history:  □ Yes □ No | |
| Allergies to medications recorded:  □ Yes □ No | | | Comment on feeding history:  □ Yes □ No | |
| Record of any prior treatment:  □ Yes □ No | | | Record of immunization history:  □ Yes □ No | |
| If treatment recorded, tick all that apply:    □ Chloroquine □ Coartem □ Amoxacillin □ Other:_____________________________________  □ SP (Fansidar) □ AQ+AS □ Septrin □ Other:_____________________________________  □ Homapak □ Quinine □ Chloramphenicol □ Other:_____________________________________ | | | | |

***Section 2: Physical examination***

| **Is the physical exam documented in the medical record?**  *Please indicate whether the physical exam is recorded, not whether the findings were normal or abnormal.* | | | |
| --- | --- | --- | --- |
| **Exam finding** | **Recorded?** | **Exam finding** | **Recorded?** |
| Temperature | □ Yes □ No | Evidence of dehydration | □ Yes □ No |
| If yes, record temperature | \|_____\|_____\| •\|_____\| ° C | Evidence of weakness | □ Yes □ No |
| Weight | □ Yes □ No | Inability to sit or stand | □ Yes □ No |
| If yes, record weight | \|_____\|_____\| kg | Comment on mental status | □ Yes □ No |
| Respiratory rate | □ Yes □ No | Comment on neck stiffness | □ Yes □ No |
| Pulse rate | □ Yes □ No | Comment on Kernig’s sign | □ Yes □ No |
| Comment on nasal flaring | □ Yes □ No | Chest exam | □ Yes □ No |
| Evidence of pallor/anemia | □ Yes □ No | Comment on chest indrawing | □ Yes □ No |
| Evidence of jaundice | □ Yes □ No | Abdominal exam | □ Yes □ No |

***Section 3: Investigations***

| **Are laboratory and radiology investigations documented in the medical record?**  *Please indicate whether the tests were ordered, and if results were recorded, not if the tests were abnormal.* | | | | |
| --- | --- | --- | --- | --- |
| **Investigation** | **Ordered?** | **Investigation** | **Recorded?** | **What was the result?** |
| Blood smear | □ Yes □ No | If BS ordered, is result recorded? | □ Yes □ No |  |
| Hemoglobin (Hb) | □ Yes □ No | If Hb ordered, is result recorded? | □ Yes □ No |  |
| Complete blood count | □ Yes □ No | If CBC ordered, is result recorded? | □ Yes □ No |  |
| Glucose | □ Yes □ No | If glucose ordered, is result recorded? | □ Yes □ No |  |
| Lumbar puncture | □ Yes □ No | If LP ordered, is result recorded? | □ Yes □ No |  |
| Chest X-ray | □ Yes □ No | If CXR ordered, is result recorded? | □ Yes □ No |  |
| Other X-ray | □ Yes □ No | If Xray ordered, is result recorded? | □ Yes □ No |  |

***Section 4: Impression***

| **Is the impression of the suspected diagnosis documented in the medical record?**  *What diagnoses were recorded? Tick all that apply.* | | | |
| --- | --- | --- | --- |
| **Impression** | **Recorded?** | **Impression** | **Recorded?** |
| Malaria | □ Yes □ No | Pneumonia | □ Yes □ No |
| Severe malaria | □ Yes □ No | Diarrhea / Dysentery | □ Yes □ No |
| Cerebral malaria | □ Yes □ No | Malnutrition / PEM | □ Yes □ No |
| Severe anemia | □ Yes □ No | Measles | □ Yes □ No |
| Meningitis | □ Yes □ No | Other: | □ Yes □ No |
| Final diagnosis on face sheet | □ Yes □ No | If yes, please indicate Dx: |  |
| Final diagnosis in notes | □ Yes □ No | If yes, please indicate Dx: |  |
| Outcome / disposition | □ Yes □ No | If yes, please indicate: | □ Discharged □ Died  □ Ran away |

***Section 5: Treatment***

| **Is the treatment plan documented in the medical record?**  *What medications were ordered? Tick all that apply.* | | | |
| --- | --- | --- | --- |
| **Medication** | **Ordered?** | **Medication** | **Ordered?** |
| Penicillin / PCN | □ Yes □ No | Coartem | □ Yes □ No |
| Chloramphenicol | □ Yes □ No | IV fluids | □ Yes □ No |
| Gentamicin | □ Yes □ No | Dextrose | □ Yes □ No |
| Ceftriaxone | □ Yes □ No | Blood transfusion | □ Yes □ No |
| Quinine IV or IM | □ Yes □ No | Nasogastric tube | □ Yes □ No |
| Quinine tablets | □ Yes □ No | Other | □ Yes □ No |
| **If antimalarial treatment ordered, record complete dosing schedule prescribed:** | | | |
