# Supplementary material for: Case Management of Severe Malaria - A Forgotten Practice: Experiences from Health Facilities in Uganda
Source: PLoS One. 2011 Mar 1;6(3):e17053. doi: 10.1371/journal.pone.0017053 (PMC3046961; doi:10.1371/journal.pone.0017053)
Supplement: Appendix S3 — Severe malaria survey tool for Inpatient unit/ward. (DOCX) [file pone.0017053.s003.docx]

**Appendix S3: Severe malaria survey tool** **For Inpatient unit/ward**

***Instructions***

*1. Complete the blank space with the answers given*

*2. Select the most appropriate option by clearly ticking the correct one/s with a pencil.*

*3. Do not prompt with the listed answers unless prompting is specified*

*4. If the Health centre III does not have admission facilities then use the form for outpatients*

**A. Geographic, Historical and Demographic information (GHD)**

1. Name of health facility: ________________________________

2. Cadre to be interviewed:

i. Nursing Aid / Asst

ii. Clinical Officer

iii. Nursing Officer

iv. Enrolled Nurse

v. Midwife only

vi. Comprehensive nurse

vii. MO

viii. SMO

ix. Consultant

x. Other ______________

3. Duration you have been at current post:

i. < 6 mths ii. 6 – 12 mths iii. > 12 mths


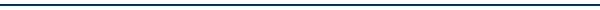


**B. Knowledge on severe malaria and its management (KW)**

1. Ask to list the **types** of severe malaria *(tick those mentioned without prompting)*

i. Cerebral malaria

ii. Severe anaemia

iii. Renal failure

iv. Pulmonary oedema

v. Hypoglycaemia

vi. Shock

vii. Spontaneous bleeding

viii. Repeated convulsions

ix. Acidosis

x. Haemoglobinuria

2. Commonest manifestation admitted in the last week [ ]

3. Ask to list **danger signs** that indicate the need for urgent attention in a very sick patient *(tick those mentioned without prompting)*

i. Rapid breathing [ ]

ii. Deep breathing [ ]

iii. Chest indrawing [ ]

iv. Unable to localise painful stimuli [ ]

v. Extreme generalised body weakness / cannot feed [ ]

vi. Convulsions / fits [ ]

vii. Very pale mucous membranes / palms [ ]

viii. Yellowing of the white part of the eyes [ ]

ix. Body temperature above 39.5^o^C [ ]

x. Has sunken eyes / fontanelle [ ]

xi. Has reduced skin turgor [ ]

xii. Repeated vomiting [ ]

xiii. Does not know any [ ]

4. Which of the following practices are important in **saving the lives** of patients with severe malaria

| Rate from 1-5 as below |  |
| --- | --- |
| Not important practice for saving life | 1 |
| Important practice for saving life | 2 |
| Very important practice for saving life | 3 |
| No idea / No response | 4 |
| Not applicable | 5 |

i. Take a long and detailed history [ ]

ii. Carry out a short but thorough examination [ ]

iii. Measure and monitor respiratory rate in those under 5 years [ ]

iv. Measure and monitor body temperature [ ]

v. Tepid sponging if febrile [ ]

vi. Fanning if febrile [ ]

vii. Measure blood glucose if unconscious [ ]

viii. Identify patients with danger signs from other patients [ ]

ix. Start prompt treatment with IV quinine [ ]

x. Do blood slide to confirm malaria parasites in blood [ ]

xi. Nurse in the lateral positions if unconscious [ ]

xii. Blood transfusion for those with severe anaemia [ ]

xiii. Educate attendants on bednets before starting treatment [ ]

5. Are there other conditions that can present like severe malaria ( Y / N )

6. If Y, which ones occur in your unit?

i. _________________

ii. ________________

iii. ________________

iv. ________________


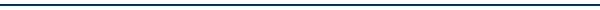


**C. Diagnosis and Treatment (DT)**

1. Do you usual make a **final** diagnosis based on:

i. clinical features only (presumptive) [ ]

ii. clinical features and diagnostic tests (confirmatory) [ ]

2. What antimalarial drugs do you **routinely** give severe malaria cases (as treatment)

i. Quinine

ii. Chloroquine

iii. Artemether

iv. Artemether-lumefantrine

v. Amodiaquine

vi. Artesunate

vii. Chloroquine / SP tablets

viii. Other _______________

xi. Referred without treatment

3. What route do you routinely use to give the antimalarial

i. IM injection

ii. IV infusion

iii. Oral tablets or syrup

iv. Rectal

v. Not applicable

4. Are children weighed before an antimalarial is prescribed? ( Y / N )

5. A patient presents to you with history of fever for 4 days, associated with convulsions and now is unconscious. You think this patient has severe malaria. What antimalarial treatment will you give this patient?

i. Quinine (Y/N)

ii. Artemether (Y/N)

iii. Artesunate (Y/N)

iv. Others, specify______________________________________________

6. For how long will you administer the antimalarial for?_________________

7. If this patient was a 4 year old child, write the exact prescription of the antimalarial you would prescribe

________________________________________________________________

7. If this patient was an adult, write the exact prescription of the antimalarial you would prescribe

_________________________________________________________________

8. What additional supportive treatment would you give to these patients?

i.__________________________________________________________

ii.__________________________________________________________

iii.__________________________________________________________

iv.__________________________________________________________

v.__________________________________________________________

9. What problems do you face in managing severe malaria cases?

a) _________________________

b) _________________________

c) _________________________

d) _________________________

10. Have you had the opportunity to improve your skills at malaria case management in the last 12 months? ( Y / N )

11. If Y, how?

i. At workshops

ii. Teaching by a colleague/senior from within the health facility

iii. Teaching by someone from outside the health facility

iv. Reading printed material / self teaching

v. Other, specify __________________________

12. Have you ever undergone IMCI training? ( Y / N )


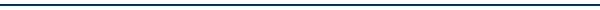


**D. Stock (ST)**

*Complete the checklist for supplies and equipment*

1. How often did you get stock outs lasting for more than one week of the items listed below in the previous three months in your unit?

| Code |  |
| --- | --- |
| Not available (but should be) | 0 |
| Available and never out-of-stock | 1 |
| 1-2 stock-outs | 2 |
| 3-4 stock-outs | 3 |
| More than 4 stock-outs | 4 |
| Not applicable | 5 |

| **Items** | **Code** | **Main reason for stock-out** |
| --- | --- | --- |
| i. Quinine (parenteral) |  |  |
| ii. Normal saline |  |  |
| iii. 50% dextrose |  |  |
| iv. Blood for transfusion |  |  |
| v. IV giving sets |  |  |
| vi. Blood transfusion sets |  |  |
| vii. Syringes |  |  |

2. Are there particular months of the year when you are more likely to get stock-outs of:

i. Quinine inj ( Y / N )

ii. Blood for transfusion ( Y / N )

3. If Y, when? i. Quinine inj ___________________________________

ii. Blood _______________________________________


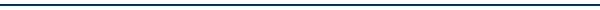


**E. Patient triage (PT)**

1. Who is usually the first to meet the patient and attendants when they arrive at the health facility?

i. 9am - Midday: _________________________________________

ii. 10pm – 1am: _________________________________________

2. Is there a method of screening very sick patients from the queue?

( Y / N )

3. If Y, who identifies them? _______________________________

______________________________________________________

4. If Y, how are they identified? ______________________________

_______________________________________________________

5. What is done for those who are screened? ___________________

________________________________________________________

6. If N, how would you want them identified? ___________________

________________________________________________________

7. If no one has the responsibility to screen very sick patients in the OPD, who do you think is the **best person** to do the screening?

_________________________________________________________


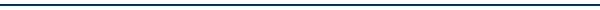


**F Timing (TI)**

1. Complete the table below using information from the health worker

| Code: |  |
| --- | --- |
| Within 30 mins | 1 |
| >30 mins – 1 hour | 2 |
| >1 hour – 3 hours | 3 |
| >3 hours | 4 |
| Not applicable | 5 |

| **Component of triage** | **9am - midday** | **10pm – 1am** |
| --- | --- | --- |
| i. Arrival to seeing the relevant health worker |  |  |
| ii. Clinical assessment to getting results of blood smear |  |  |
| iii. Clinical assessment to getting first treatment dose |  |  |
| v. Clinical assessment to getting a blood transfusion |  |  |
| vi. Getting referral note to departure from the health facility |  |  |


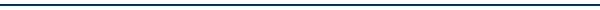


**G. Referral system (R)**

*(Do not complete this section if the health facility does not refer patients or if the interviewee is not involved in referring patients)*

1. Number of patients with severe malaria that have been referred that day

[ ]

2. What are the reasons why you decide to refer patients with severe malaria?

i. Lack of blood for transfusion at the facility (Y / N)

ii. Poor response to treatment given (Y / N)

iii. Lack of I.V fluids (Y / N)

iv. Lack of Oxygen (Y / N)

v. No beds available to admit patient (Y / N)

vi. Others, specify___________________________________________

___________________________________________________________

3. Do you use the presence of some clinical signs to make referral decisions?

(Y / N)

4. If Y, what signs do you use?

i. Rapid breathing [ ]

ii. Deep breathing [ ]

iii. Chest indrawing [ ]

iv. Unable to localise painful stimuli [ ]

v. Extreme generalised body weakness / cannot feed [ ]

vi. Convulsions / fits [ ]

vii. Very pale mucous membranes / palms [ ]

viii. Yellowing of the white part of the eyes [ ]

ix. Body temperature above 39.5^o^C [ ]

x. Has sunken eyes / fontanelle [ ]

xi. Repeated vomiting [ ]

xii. Others, specify_______________________________________

5. When you refer to another health facility do you give any pre-referral medications ( Y / N )

6. If Y, what do you give?

Generic name Route of administration

i. _________________ ___________________

ii._________________ ___________________

iii. ________________ ___________________

7. If N, why not? __________________________________________

8. Do you give a referral note? ( Y / N )

9. Where do you refer the patients to *(name)*?

i. _______________________ approx distance from unit _________ km

ii. _______________________ approx distance from unit _________ km

10. Do you give the attendants directions to get to the health facility? ( Y / N )

11. Do you tell the attendants what form of transport to use? (Y / N )

12. Do you tell them where to report when they get to the health facility?

( Y / N )

13. Do you give any other advice ( Y / N )

11. If Y, what? ____________________________________________

14. Do you have a method of finding out the outcome of the referral?

( Y / N )

15. If Y, how _____________________________________________

16. If N, would you like to know the outcome? ( Y / N )


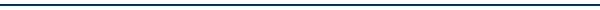


**H. Supervision on Malaria Case Management (SU)**

1. Have you undergone any form of supervision on the management of malaria in the last six months? ( Y / N )

2. If Y, were you comfortable with the process? ( Y / N )

3. Who has supervised you in the last six months?

Within the health facility

i. Colleague [ ]

ii. Immediate senior [ ]

iii. Head of unit [ ]

iv. Head of health facility [ ]

From outside the health facility

v. Malaria focal person [ ]

vi. Malaria zonal coordinator [ ]

vii. Staff from health subdistrict [ ]

viii. Consultant from the nearest referral hospital [ ]

ix. Ministry of Health technical staff [ ]

x. Health worker from abroad [ ]

4. How often have you been supervised in the last 6 months?

i. Once

ii. Twice

iii. Thrice

iv. Monthly

v. None

5. What methods have you been supervised with in the last 6 months?

i. Direct observation of care

ii. Interviews

iii. Inspection

iv. Feedback

v. Problem-solving

vi. Coaching

vii. Training

viii. Decision-making

ix. Clinical audit

x. Other, specify ______________

6. Do you feel support supervision for malaria is useful? ( Y / N )

7. If yes, how is it useful?

i. Improved competence / skills

ii. Improved compliance with national guidelines

iii. Improved care given to patients

iv. Improved motivation

v. Other, specify _____________________________________

8. Can you list any international organisations or NGOs that are involved in malaria work where your facility is located

- 1. _________________________________________
  2. _________________________________________
  3. _________________________________________

9. How have these international organisations or NGOs been useful to you or the community?

i._________________________________________

ii._________________________________________

iii._________________________________________

iv._________________________________________


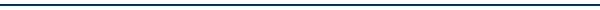


**I. Roles and Responsibilities (RR)**

1. Were you given a job description when you started your current post? ( Y / N )

2. If Yes, was it i. written or ii. verbal

3. What would you consider as your role in the routine management of patients with severe malaria?

i. _______________________________________________________

ii. ______________________________________________________

iii. ______________________________________________________


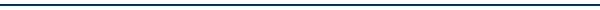


**J. Aides Memoir (AM)**

1. Which of the following severe malaria case management aides are available at the unit?

i. Posters on the wall ( Y / N )

ii. Wall charts ( Y / N )

ii. Leaflets / Pamphlets ( Y / N )

iii. Reference textbooks ( Y / N )

iv. Desk aids ( Y / N )

Others, specify _________________________________________

2. Which do you prefer as a reminder?

| Rate from 1 to 5 as below |  |
| --- | --- |
| Not useful | 1 |
| A good reminder | 2 |
| A very good reminder | 3 |
| No idea / No response | 4 |
| Not applicable | 5 |

i. Posters on the wall [ ]

ii. Wall charts [ ]

ii. Leaflets / Pamphlets [ ]

iii. Reference textbooks [ ]

iv. Desk aids [ ]

Others, specify _________________________________________


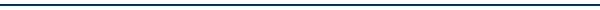


**K. Adverse reactions (AR)**

1. Do you inform attendants of the adverse reactions of the antimalarial which the patient is getting? ( Y / N )

2. If Y, which ones do you mention for quinine

Drug Adverse Reaction

Quinine i. ________________________________

ii. ________________________________

iii.________________________________

3. If N, why not_________________________________________

4. Do you record and report suspected adverse reactions of any of the drugs that you use in your facility? ( Y / N )

5. If Y, were do you record _______________________________________

6. Who do you report to __________________________________________

7. If N, why not? __________________________________________


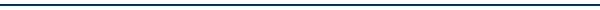


**L. Care and monitoring (CM)**

1. What cadre of staff are always available on the ward *(fill table below with the options from the key)*

| Cadre | Code |
| --- | --- |
| Nursing Aid / Asst | 1 |
| Registered Nurse | 2 |
| Enrolled Nurse | 3 |
| Clinical officer | 4 |
| Doctor | 5 |

| Period | Always available |
| --- | --- |
| i. 8am – midday |  |
| ii. 1pm – 5pm |  |
| iii. 6pm – 10pm |  |
| iv. 11pm – 3am |  |
| v. 4am – 8am |  |

2. Do you think that qualified nurses are competent enough to start patients with severe malaria on treatment with IV quinine without waiting for the clinical officer or doctor to prescribe? ( Y / N )

3. Are there occasions when you give a loading dose of quinine? ( Y / N )

4. If Y, when do you do so ___________________________________

5. If N, why not __________________________________________

6. Do you routinely monitor unconscious patients with

i. Glasgow coma scale ( Y / N )

ii. Blantyre coma scale ( Y / N )

7. How often do you measure the following parameters in patients with severe malaria on the first day of admission?

| Parameter | Frequency (hourly) |
| --- | --- |
| i. Temperature |  |
| ii. Pulse |  |
| iii. Blood pressure |  |
| iv. Respiratory rate |  |
| v. Unconsciousness |  |
| vi. Blood glucose |  |
| vii. Convulsions |  |
| viii. Haemoglobin concentration |  |
| ix. Parasitaemia |  |


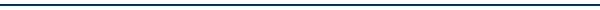


**M. Investigations (IG)**

1. List the investigations that can routinely be carried out on patients with severe malaria in your health facility?

i. _____________________________________________

ii. ____________________________________________

iii. ____________________________________________

iv. ____________________________________________

v. ____________________________________________

vi. ____________________________________________

vii. ___________________________________________

viii. ___________________________________________

2. In severe malaria patients, do you routinely repeat the blood smear for malaria parasites to monitor parasite clearance?

3. If Y, how often? _______________________________________

4. If N, why not? _________________________________________

5. Who routinely takes the blood specimens from the patients? ______________

6. Who routinely takes the specimens to the laboratory? ________________

7. Are urgent laboratory requests marked in any special way? ( Y / N )

8. If Y, in what way? _______________________________________

9. If Y, does the laboratory process them urgently? ( Y / N )

10. If Y, what is the average time to get them back? ____ (hours)

11. If N, why not _____________________________________

1. Who routinely collects the lab results from the laboratory? _________________


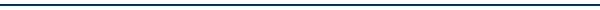


**N. Follow-up (FU)**

1. Are survivors of severe malaria followed-up? ( Y / N )

2. If Y, what assessments are carried out during follow-up?

i. Hb measurement ( Y / N )

ii. Blood smear for malaria parasites ( Y / N )

iii. Growth monitoring ( Y / N )

iv. Assessments for neurological sequelae ( Y / N )

v. Other ___________________________________________

2. How often are these patients followed-up?______________________________


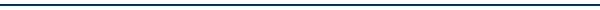


**O. Death due to severe Malaria**

1. On what days of the week do most of these deaths occur?

i. Monday to Wednesday ( Y / N )

ii. Thursday to Friday ( Y / N )

iii. Weekends ( Y / N )

2. At what times do these deaths commonly occur?

i. Mornings ( Y / N )

ii. Afternoons ( Y / N )

iii. Evenings ( Y / N )

iii. Nights

**P. Quality of care (QC)**

1. How do you rate the quality of care that your unit gives to patients with severe malaria? [ ]

| Rate from 1 to 5 as below |  |
| --- | --- |
| Poor quality | 1 |
| Good quality | 2 |
| Very good quality | 3 |
| No idea / No response | 4 |
| Not applicable | 5 |

i. Quality of diagnosis [ ]

ii. Quality of treatment [ ]

iii. Quality of nursing care [ ]

iv. Quality of supportive care [ ]

v. Quality of follow-up [ ]

vi. Quality of management of the health facility [ ]

2. What specific aspects of care are weak in your health facility?

i. ________________________________________________________

ii. ________________________________________________________

iii. _______________________________________________________

3. What specific aspects of care are done very well in your health facility?

i. ________________________________________________________

ii. ________________________________________________________

iii. _______________________________________________________

4. What suggestions do you have to improve the quality of care given to patients with severe malaria in your health facility?

i. __________________________________________________________

ii. __________________________________________________________

iii. _________________________________________________________

iv. _________________________________________________________

Date: ___ / ____ / 2009 Time _______ am /pm

Completed by: _________________ (name)
