# Supplementary material for: Case Management of Severe Malaria - A Forgotten Practice: Experiences from Health Facilities in Uganda
Source: PLoS One. 2011 Mar 1;6(3):e17053. doi: 10.1371/journal.pone.0017053 (PMC3046961; doi:10.1371/journal.pone.0017053)
Supplement: Appendix S4 — Severe malaria survey tool - Checklist for each Health Facility. (DOCX) [file pone.0017053.s004.docx]

**Appendix S4: Severe malaria survey tool - Checklist for each Health Facility**

***Instructions***

*1. Complete the blank spaces*

*2. Select the most appropriate option by clearly ticking the correct one/s with a pencil.*

*3. Complete this checklist by observing what goes on in the units of the health facility*

**A. Geographic, Historical and Demographic information (GDC)**

1. Name of health facility: ________________________________

2. Grade of health facility

i. HC II

ii. HC III

iii. HC IV

iv. District Hospital

v. Other ______________

3. Type of health facility

i. Government facility

ii. Faith-based facility

iii. Private-for-profit based facility

iv. Other __________________

4. Name of village: _________________ 5. Parish: __________________

6. Subcounty: _____________________ 7. District: __________________

8. Approximate size of population in catchment area __________________


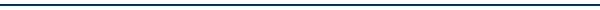


**B. HUMAN RESOURCES (HRS)**

Record the number of personnel by cadre, carefully recording the following information:

- Number of staff employed in the facility
- Number of staff scheduled to be on duty on the day of survey
- Number of staff present during the survey

| **Cadre** | **Number required according to MOH staffing norms** | **Number of staff employed in facility** | **Number of staff scheduled for duty today** | **Number of staff present on duty today** |
| --- | --- | --- | --- | --- |
| Medical Doctor |  |  |  |  |
| Health officer |  |  |  |  |
| Clinical Nurse |  |  |  |  |
| Public Health Nurse |  |  |  |  |
| Midwife |  |  |  |  |
| Comprehensive Nurse |  |  |  |  |
| Community health worker |  |  |  |  |
| Nursing Aids |  |  |  |  |
| Laboratory technicians |  |  |  |  |
| Nursing Aids |  |  |  |  |
| Other (Specify) |  |  |  |  |


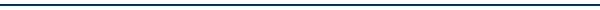


**C. Records (RCC)**

1. Is there a register for keeping record of patients seen in OPD ( Y / N )

2. If Y, is it uptodate (by yesterday) ( Y / N )

3. Is there a register for keeping record of patients admitted ( Y / N / NA)

4. If Y, is it uptodate (by yesterday) ( Y / N )

5. Do the records note:

i. Age of patient ( Y / N )

ii. Type of severe malaria manifestation ( Y / N )

iii. If microscopy was performed ( Y / N )

iii. Records are not clear

6. In April 2009, what was the number of severe malaria cases?

i. Referred _________________ or Not applicable

ii. Admitted _________________ or Not applicable

iii. Died in health facility _______________ or Not applicable

8. Of the patients admitted with severe malaria last year, how many died? (Also mention the total number admitted with severe malaria during this period)

i. Number of adults__________________________________

ii. Number of children________________________________

9. Of the patients admitted with severe malaria last month, how many died? (Also mention the total number admitted with severe malaria during this period)

i. Number of adults__________________________________

ii. Number of children________________________________

10. What are the common causes of death in patients presenting with severe malaria at this health facility

1. Cerebral malaria (Y/N)

2. Severe anaemia (Y/N)

3. Hypoglycaemia (Y/N)

4. Severe dehydration (Y/N)

5. Respiratory distress (Y/N)

6. Others, specify________________________________

11. How are records of death kept in this facility?

1. Inpatient register

2. Death register

3. Other, specify____________________________________

12. Were source documents of death records verified by the interviewer? (Y/N)

13. If yes, comment on the quality of records

1. Good quality

2. Poor quality

3. Accurate

4. Inaccurate/incomplete

5. Other, specify__________________________________________________


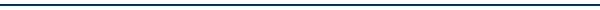


**D. Supplies and Equipment (SAEC)**

1. Which of the following diagnostic facilities are available and functional **within** the unit specified *(A=available, F=functional, AF=available and functional, N=None, use these letters to indicate the pertaining situation)*

| **Test** | **OPD** | **Children ward** |
| --- | --- | --- |
| i. No diagnostic facilities |  |  |
| ii. Malaria Rapid test kit |  |  |
| iii. Parasight F |  |  |
| iv. ParaCheck |  |  |
| v. Optimal |  |  |
| vi. Hand-held Glucometer |  |  |
| vii. Glucose dipstick |  |  |
| viii. Urine dipstick |  |  |
| ix. Hb colou  r scale |  |  |
| x. HemoCue™ haemoglobinometer |  |  |
| xi. Microscopy |  |  |

2. Complete this table for the OPD noting the supplies that the staff in the OPD currently have **access to**.

|  | **Item** | **Specification** | **√/×** | **NA** |
| --- | --- | --- | --- | --- |
|  | **Drugs** |  |  |  |
| 1 | Quinine | Injectable |  |  |
| 2 |  | Oral |  |  |
| 3 | Chloroquine | Injectable |  |  |
| 4 | Sulphadoxine-pyrimethamine | Oral |  |  |
| 5 | Artemether-lumefantrine | Oral |  |  |
| 6 | Artemether | Injectable |  |  |
| 7 | Artemisinin | Rectal |  |  |
| 8 | Artesunate | Iv |  |  |
| 9 |  | Rectal |  |  |
| 10 | Arteether | Injectable |  |  |
| 11 | Diazepam | Injectable |  |  |
| 12 |  | Rectal |  |  |
| 13 | Dextrose | 50% |  |  |
| 14 |  | 30% |  |  |
| 15 |  | 25% |  |  |
| 16 | Paracetamol | Oral |  |  |
| 17 |  | suppositories |  |  |
| 18 | Phenobarbitone | Injection |  |  |
| 19 | Furosemide | Injection |  |  |
|  | **Item** | **Specification** | **√/×** | **NA** |
|  | **Fluids** |  |  |  |
| 20 | Dextrose | 5% |  |  |
| 21 |  | 10% |  |  |
| 22 |  | 50% |  |  |
| 23 | Saline | 0.9% |  |  |
| 24 | Darrow’s solution | Half strength |  |  |
| 25 |  | Full strength |  |  |
| 26 | Ringer lactate | 500ml |  |  |
| 27 | Fluid bottles | 100ml |  |  |
| 28 |  | 200ml |  |  |
| 29 |  | 500ml |  |  |
| 30 | Water for injection |  |  |  |
|  | **Medical** |  |  |  |
| 31 | NG tube | Paediatric sizes |  |  |
| 32 |  | Adult sizes |  |  |
| 33 | IV giving sets |  |  |  |
| 34 | Blood transfusion sets |  |  |  |
| 35 | IV cannulae | Paediatric sizes |  |  |
| 36 |  | Adult sizes |  |  |
| 37 | Scalp vein butterfly needles |  |  |  |
| 38 | Needles disposable |  |  |  |
| 39 | Syringes | 2ml |  |  |
| 40 |  | 5ml |  |  |
| 41 |  | 10ml |  |  |
| 42 |  | 20ml |  |  |
| 43 | Syringe feeding | 50/60ml |  |  |
| 44 | Gloves | Sterile |  |  |
| 45 |  | Disposable |  |  |
| 46 | Cotton wool |  |  |  |
| 47 | Adhesive tape |  |  |  |
| 48 | Lancets |  |  |  |
| 49 | Oxygen in cylinders |  |  |  |
|  | **Equipment** |  |  |  |
| 50 | Thermometer |  |  |  |
| 51 | Weighing scale | Hanging/Salter |  |  |
| 52 |  | Electronic |  |  |
| 53 |  | Bathroom |  |  |
| 54 |  | Other |  |  |
| 55 | Examination table |  |  |  |
| 56 | Stethoscope |  |  |  |
| 57 | Clock/Watch |  |  |  |
| 58 | BP machine |  |  |  |
| 59 | Ophthalmoscope |  |  |  |
| 60 | Otoscope |  |  |  |
| 61 | Oral airways |  |  |  |
| 62 | Ambubag |  |  |  |
|  | **Item** | **Specification** | **√/×** | **NA** |
| 63 | Torch |  |  |  |
| 64 | Glucometer |  |  |  |
| 65 | Glucose dipsticks |  |  |  |
| 66 | Urine dipsticks |  |  |  |

3. Complete this Table for the children's ward noting the supplies that the staffs there currently have **access to**.

|  | | **Item** | | **Specification** | | **√/×** | | **NA** |
| --- | --- | --- | --- | --- | --- | --- | --- | --- |
|  | | **Drugs** | |  | |  | |  |
| 1 | | Quinine | | Injectable | |  | |  |
| 2 | |  | | Oral | |  | |  |
| 3 | | Chloroquine | | Injectable | |  | |  |
| 4 | | Sulphadoxine-pyrimethamine | | Oral | |  | |  |
| 5 | | Artemether-lumefantrine | | Oral | |  | |  |
| 6 | | Artemether | | Injectable | |  | |  |
| 7 | | Artemisinin | | Rectal | |  | |  |
| 8 | | Artesunate | | Iv | |  | |  |
| 9 | |  | | Rectal | |  | |  |
| 10 | | Arteether | | Injectable | |  | |  |
| 11 | | Diazepam | | Injectable | |  | |  |
| 12 | |  | | Rectal | |  | |  |
| 13 | | Dextrose | | 50% | |  | |  |
| 14 | |  | | 30% | |  | |  |
| 15 | |  | | 25% | |  | |  |
| 16 | | Paracetamol | | Oral | |  | |  |
| 17 | |  | | suppositories | |  | |  |
| 18 | | Phenobarbitone | | Injection | |  | |  |
| 19 | | Furosemide | | Injection | |  | |  |
|  | | **Fluids** | |  | |  | |  |
| 20 | | Dextrose | | 5% | |  | |  |
| 21 | |  | | 10% | |  | |  |
| 22 | | Saline | | 0.9% | |  | |  |
| 23 | | Fluid bottles | | 100ml | |  | |  |
| 24 | |  | | 200ml | |  | |  |
| 25 | |  | | 500ml | |  | |  |
| 26 | | Darrow’s solution | | Half strength | |  | |  |
| 27 | |  | | Full strength | |  | |  |
| 28 | | Ringer lactate | |  | |  | |  |
| 29 | | Water for injection | |  | |  | |  |
| 30 | | Blood for transfusion | | Packed cells | |  | |  |
| 31 | |  | | Whole blood | |  | |  |
|  | **Item** | | **Specification** | | **√/×** | | **NA** | |
|  | **Medical** | |  | |  | |  | |
| 32 | NG tube | | Paediatric sizes | |  | |  | |
| 33 |  | | Adult sizes | |  | |  | |
| 34 | IV giving sets | |  | |  | |  | |
| 35 | Blood transfusion sets | |  | |  | |  | |
| 36 | IV cannulae | | Paediatric sizes | |  | |  | |
| 37 |  | | Adult sizes | |  | |  | |
| 38 | Scalp vein butterfly needles | |  | |  | |  | |
| 39 | Needles disposable | |  | |  | |  | |
| 40 | Syringes | | 2ml | |  | |  | |
| 41 |  | | 5ml | |  | |  | |
| 42 |  | | 10ml | |  | |  | |
| 43 |  | | 20ml | |  | |  | |
| 44 | Syringe feeding | | 50/60ml | |  | |  | |
| 45 | Lumbar puncture needles | | Paediatric sizes | |  | |  | |
| 46 |  | | Adult sizes | |  | |  | |
| 47 | Intraosseous needles | | Paediatric sizes | |  | |  | |
| 48 |  | | Adult sizes | |  | |  | |
| 49 | Gloves | | Sterile | |  | |  | |
| 50 |  | | Disposable | |  | |  | |
| 51 | Urinary catheters Foley | | Paediatric sizes | |  | |  | |
| 52 |  | | Adult sizes | |  | |  | |
| 53 | Urinary catheters condom | | Small size | |  | |  | |
| 54 |  | | Medium size | |  | |  | |
| 54 |  | | Large size | |  | |  | |
| 56 | Suction catheters | | Paediatric | |  | |  | |
| 57 |  | | Adult | |  | |  | |
| 58 | Cotton wool | |  | |  | |  | |
| 59 | Adhesive tape | |  | |  | |  | |
| 60 | Lancets | |  | |  | |  | |
|  | **Equipment** | |  | |  | |  | |
| 61 | Thermometer | |  | |  | |  | |
| 62 | Weighing scale | | Hanging/Salter | |  | |  | |
| 63 |  | | Electronic | |  | |  | |
| 64 |  | | Bathroom | |  | |  | |
|  |  | |  | |  | |  | |
| 65 | Examination table | |  | |  | |  | |
| 66 | Stethoscope | |  | |  | |  | |
| 67 | Clock/Watch | |  | |  | |  | |
| 68 | BP machine | |  | |  | |  | |
| 69 | Ophthalmoscope | |  | |  | |  | |
| 70 | Otoscope | |  | |  | |  | |
| 71 | Oral airways | |  | |  | |  | |
| 72 | Ambubag | |  | |  | |  | |
| 73 | Oxygen | | Cylinders | |  | |  | |
| 74 |  | | Dispenser | |  | |  | |
|  | **Item** | | **Specification** | | **√/×** | | **NA** | |
| 75 | Suction machine | |  | |  | |  | |
| 76 | Torch | |  | |  | |  | |
| 77 | Glucometer | |  | |  | |  | |
| 78 | Glucose dipsticks | |  | |  | |  | |
| 79 | Urine dipsticks | |  | |  | |  | |
| 80 | HB colour scale | |  | |  | |  | |
| 81 | HemoCue/Haemoglobinometer | |  | |  | |  | |
| 82 | Specimen bottles/vacutaniers | | EDTA | |  | |  | |
| 83 |  | | Plain | |  | |  | |
| 84 |  | | Sodium citrate | |  | |  | |
| 85 |  | | Clot activator | |  | |  | |
| 86 | Microscope slides | |  | |  | |  | |


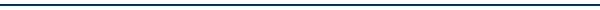


**E. Patient triage (PTC)**

1. Is there a defined triage system in place *(observation)*? ( Y / N )

2. If N, why not? __________________________________________

________________________________________________________

3. What is the entry point to the facility ___________________________________

4. Visible directions tell people where to go ( Y / N )

5. Screening of sick patients at OPD queue ( Y / N / NA )

6. Separate lines for children and adults at OPD queue ( Y / N / NA )

7. Screening of sick patients for urgent treatment on admission queue ( Y / N / NA )

8. Urgent attention given to sick patients at OPD queue ( Y / N / NA )

9. Urgent attention given to sick patients on admission queue ( Y / N / NA )

10. Lab requests marked for urgent response ( Y / N / NA )

11. Lab results that are urgent are given priority ( Y / N / NA )

12. Lab results that are urgent are returned to requester as priority ( Y / N / NA )

13. Describe any other features not captured above _________________________

___________________________________________________________________


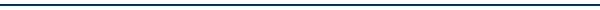


**F. Aides Memoir (AMC)**

1. Which of the following severe malaria case management aides are located in **visible** areas for the staff in the **OPD**?

i. Posters on the wall ( Y / N )

ii. Wall charts ( Y / N )

ii. Leaflets / Pamphlets ( Y / N )

iii. Reference textbooks ( Y / N )

iv. Desk aids ( Y / N )

Others, specify _________________________________________

2. Which of the following severe malaria case management aides are located in **visible** areas for the staff at the **children’s ward**?

i. Posters on the wall ( Y / N )

ii. Wall charts ( Y / N )

ii. Leaflets / Pamphlets ( Y / N )

iii. Reference textbooks ( Y / N )

iv. Desk aids ( Y / N )

Others, specify _________________________________________


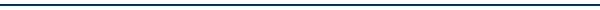


**G. Communication (COC)**

1. Is there an easy and quick means of communicating with the other departments within the health facility ( Y / N )

2. If Y, which forms of communication exist

i. Direct communication in a small unit [ ]

ii. Telephone [ ]

iii. Other_____________________________

3. Is there a means of communicating with the other health facilities in the district?

( Y / N )

4. If Y, which forms of communication exist

i. Radio [ ]

ii. Telephone [ ]

iii. Other_____________________________

5. Are there regular meetings with other staff in the facility? ( Y / N )

6. If Y, how often? ___________________________


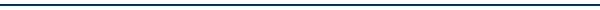


**H. Quality of care (QCC)**

1. How do you rate the quality of care that the unit gives to patients with severe malaria? [ ]

| Rate from 1 to 5 as below |  |
| --- | --- |
| Poor quality | 1 |
| Good quality | 2 |
| Very good quality | 3 |
| No idea / No response | 4 |
| Not applicable | 5 |

i. Quality of diagnosis [ ]

ii. Quality of treatment [ ]

iii. Quality of nursing care [ ]

iv. Quality of supportive care [ ]

v. Quality of follow-up [ ]

vi. Quality of management of the health facility [ ]

2. What specific aspects of care are weak in the health facility?

i. ________________________________________________________

ii. ________________________________________________________

iii. _______________________________________________________

3. What specific aspects of care are done very well in the health facility?

i. ________________________________________________________

ii. ________________________________________________________

iii. _______________________________________________________

Date: ___ / ____ / 2009 Time _______ am /pm

Completed by: _________________ (name)
