# Supplementary material for: Case Management of Severe Malaria - A Forgotten Practice: Experiences from Health Facilities in Uganda
Source: PLoS One. 2011 Mar 1;6(3):e17053. doi: 10.1371/journal.pone.0017053 (PMC3046961; doi:10.1371/journal.pone.0017053)
Supplement: Appendix S5 — Severe malaria survey tool for pharmacy. (DOCX) [file pone.0017053.s005.docx]

# Appendix S5: Severe malaria survey tool for pharmacy

***Instructions***

*1. Complete the blank space with the answers given*

*2. Select the most appropriate option by clearly ticking the correct one/s with a pencil.*

*3. Do not prompt with the listed answers unless prompting is specified*

**A. Geographic, Historical and Demographic information (GHD)**

1. Name of health facility: ________________________________

2. Cadre to be interviewed: ______________________________________

3. Duration you have been at current post:

i. < 6 mths ii. 6 – 12 mths iii. > 12 mths

4. Any previous history of training on supply chain management of drugs ( Y / N )


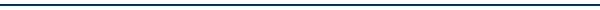


**B. Records (RC)**

1. Register for keeping record of antimalarial usage ( Y / N )

2. If Y, are they uptodate (yesterday) ( Y / N )


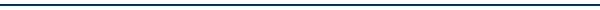


**C. Stock (SK)**

*Complete the checklist for supplies and equipment*

1. How often did you get stock outs lasting more than one week of the items listed below in the previous three months in your unit?

| Code |  |
| --- | --- |
| Not available (but should be) | 0 |
| Available and never out-of-stock | 1 |
| 1-2 stock-outs | 2 |
| 3-4 stock-outs | 3 |
| More than 4 stock-outs | 4 |
| Not applicable | 5 |

| **Items** | **Frequency** | **Main reason for stock-out** |
| --- | --- | --- |
| i. Quinine (parenteral) |  |  |
| ii. Normal saline |  |  |
| iii. 50% dextrose |  |  |
| iv. 5% dextrose |  |  |
| v. Blood for transfusion |  |  |
| vi. IV giving sets |  |  |
| vii. Blood transfusion set |  |  |
| viii. Syringes |  |  |
| ix. Quinine tablets |  |  |

2. Are there particular months of the year when you are more likely to get stock-outs of:

i. Quinine inj ( Y / N )

ii. Blood for transfusion ( Y / N )

iii. Quinine tablets ( Y / N )

3. If Y, when? i. Quinine inj ___________________________________

ii. Blood _______________________________________

iii. Quinine tablets _______________________________


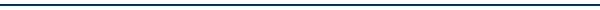


**D. Supplies and Supply management (SSM)**

1. Which of the following are available in the pharmacy unit?

| **Item** | **Specification** | **Yes** | **No** |
| --- | --- | --- | --- |
| **Drugs** |  |  |  |
| Quinine | Injectable |  |  |
|  | Oral |  |  |
| Chloroquine | Injectable |  |  |
| Sulphadoxine-pyrimethamine | Oral |  |  |
| Artemether-lumefantrine | Oral |  |  |
| Artemether | Injectable |  |  |
| Artemisinin | Rectal |  |  |
| Artesunate | IV |  |  |
|  | Rectal |  |  |
| Arteether | Injectable |  |  |
| Diazepam | Injectable |  |  |
|  | Rectal |  |  |
| Paracetamol | Oral |  |  |
|  | Suppositories |  |  |
| Phenobarbitone | Injection |  |  |
| Furosemide | Injection |  |  |
| **Fluids** |  |  |  |
| Dextrose | 50% |  |  |
|  | 30% |  |  |
|  | 25% |  |  |
|  | 10% |  |  |
|  | 5% |  |  |
| Saline | 0.9% |  |  |
| Fluid bottles | 100ml |  |  |
|  | 200ml |  |  |
|  | 500ml |  |  |
| Darrow’s solution | Half strength |  |  |
| **Item** | **Specification** | **Yes** | **No** |
|  | Full strength |  |  |
| Ringer lactate |  |  |  |
| Water for injection |  |  |  |

2. Is there a method in place for preventing stocks-outs? ( Y / N )

3. If Y, what is done? _____________________________________

________________________________________________________

4. If N, why not? __________________________________________

________________________________________________________

5. In the last year has quinine expired in the pharmacy/store? ( Y / N )

6. If Y, why? ________________________________________

7. Is there a method in place for quantifying the antimalarial needs? ( Y / N )

8. If Y, what is it? ________________________________________

________________________________________________________

9. If N, why not? _________________________________________

10. Is your supply of antimalarials sufficient for the patients that are admitted? ( Y / N )

11. If N, which ones are not? ______________________________

12. If Y, how do you ensure adequate supply? _________________

______________________________________________________

13. Do you supply IV fluids in smaller bottles (100ml/200ml) for children ( Y / N )

14. If Y, what have been the benefits _________________________

_______________________________________________________

15. If N, why not? _________________________________________

________________________________________________________

16. Do you keep oxygen for use on the inpatient medical and paediatric wards ( Y / N )

17. If N, why not? ________________________________________

17. What specific aspects of drug management and supply are weak in your health facility?

i. ________________________________________________________

ii. ________________________________________________________

iii. _______________________________________________________

18. What specific aspects of drug management and supply do you think are performed very well in your health facility?

i. ________________________________________________________

ii. ________________________________________________________

iii. _______________________________________________________

19. What suggestions do you have to improve the quality of treatment given to patients with severe malaria in your health facility?

i. __________________________________________________________

ii. __________________________________________________________

iii. _________________________________________________________

iv. _________________________________________________________


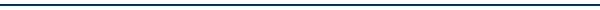


**E. Supervision (SUp)**

1. Have you undergone any form of supervision in the last six months? ( Y / N )

2. If Y, were you comfortable with the process? ( Y / N )

3. Who has supervised you in the last six months?

Within the health facility

i. Colleague

ii. Immediate senior

iii. Head of unit

iv. Head of health facility

From outside the health facility

Specify _________________________

4. How often have you been supervised in the last six months?

i. Once

ii. Twice

iii. Thrice

iv. Monthly

v. None


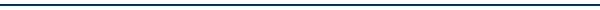


**F. Pharmacovigilance (PV)**

1. Are you informed of any adverse reactions of the drugs that are used in your facility? ( Y / N )

2. If Y, which drugs do you routinely keep records for their reactions? ________________________________________________________

________________________________________________________

3. If Y, who informs you and how? ____________________________

7. If Y, who do you report them to? ___________________________

________________________________________________________

8. If N, why not? __________________________________________

Date: ___ / ____ / 2009 Time _______ am /pm

Completed by: _________________ (name)
