# Supplementary material for: Case Management of Severe Malaria - A Forgotten Practice: Experiences from Health Facilities in Uganda
Source: PLoS One. 2011 Mar 1;6(3):e17053. doi: 10.1371/journal.pone.0017053 (PMC3046961; doi:10.1371/journal.pone.0017053)
Supplement: Appendix S6 — Severe malaria survey tool for laboratory. (DOCX) [file pone.0017053.s006.docx]

# Appendix S6: Severe malaria survey tool for laboratory

***Instructions***

*1. Complete the blank space with the answers given*

*2. Select the most appropriate option by clearly ticking the correct one/s with a pencil.*

*3. Do not prompt with the listed answers unless prompting is specified*

**A. Geographic, Historical and Demographic information (GHD)**

1. Name of health facility: ________________________________

2. Cadre to be interviewed:

i. Laboratory assistant

ii. Lab technician

iii. Lab technologist

iv. Senior lab technologist

v. Microscopist

vi. Other _______________

3. Duration you have been at current post:

i. < 6 mths ii. 6 – 12 mths iii. > 12 mths

4. Any training on malaria laboratory diagnosis in the last 12 months ( Y / N )


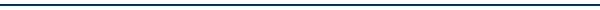


**B. Records (RC)**

1. Register for keeping record of patients investigated ( Y / N )

2. If Y, are they uptodate (yesterday) ( Y / N )

4. Do the records note the:

i. Age of patient ( Y / N )

ii. Type of severe malaria manifestation ( Y / N )

iii. Records are not clear


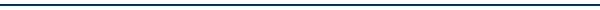


**C. Laboratory diagnosis (LD)**

1. Which of the following diagnostic investigations are done in your laboratory?

| **Investigation** | **( √ ∕ × )** |
| --- | --- |
| Blood smear thick film |  |
| Blood smear thin film |  |
| Blood smear parasite count |  |
| Malaria Rapid test kit |  |
| Hb estimation – Sahl’s method |  |
| Hb estimation – Hb colour scale |  |
| HB estimation - HemoCue™ haemoglobinometer |  |
| Glucose blood concentration – Portable glucometer |  |
| Glucose blood concentration – Glucose-oxidase method |  |
| Glucose blood concentration – Glucostick method |  |
| White blood cell count – total |  |
| White blood cell count – differential |  |
| Red blood cell count |  |
| Platelet count |  |
| Haematocrit |  |
| Blood film |  |
| ESR |  |
| CSF analysis – glucose concentration |  |
| CSF analysis – protein concentration |  |
| CSF analysis – white cell count |  |
| CSF analysis – Gram stain |  |
| CSF analysis – India ink |  |
| CSF analysis – ZN stain |  |
| CSF analysis – culture and sensitivity |  |
| Urinalysis – dipstick |  |
| Serum electrolytes |  |
| Sickling test |  |
| HIV serology |  |
|  |  |

2. Are there any other diagnostic investigations not listed above? ( Y / N )

3. If Y, which ones? _______________________________________

4. In patients with malaria admitted to the wards, are you **routinely** asked to repeat the blood smear for malaria parasites to monitor parasite clearance?

( Y / N )

5. If Y, how often __________________________________________

6. If N, why not? __________________________________________

7. Who **routinely** brings the blood specimens to the laboratory *(how are the blood specimens collected)*? ___________________________________________

8. Are urgent laboratory requests marked in any **special** way? ( Y / N )

9. If Y, in what way? _______________________________________

10. If Y, does the laboratory process them **urgently**? ( Y / N )

11. If Y, what is the average time to get urgent results back to the ward? ______ hrs _______ mins

12. If N, why not _____________________________________

13. Do you have a designated area in the lab where urgent specimens are put? ( Y / N )

14. Where are the results put once investigations have been done?

i. Non-urgent results ______________________________________

ii. Urgent results _________________________________________

15. How do the results get back to the wards? _______________________

16. Do you have a **working** microscopy? ( Y / N )

17. If Y, is it i. mono-ocular or ii. bi-ocular

18. If Y, do you use i. electricity ii. sunlight or iii. both

19. If Y, what quality control measures do you have to make sure that

the results of microscopy are accurate and up to standard on a **regular**

basis? i. __________________________________________

ii. __________________________________________

iii. __________________________________________

20. Do you have the opportunity to discuss your laboratory results with the clinicians on the wards? ( Y / N / NA )


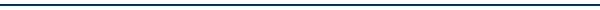


**D. Supervision on Malaria laboratory diagnosis (SUL)**

1. Have you undergone any form of supervision on malaria laboratory diagnosis in the last six months? ( Y / N )

2. If Y, were you comfortable with the process? ( Y / N )

3. Who has supervised you in the last six months?

Within the health facility

i. Colleague

ii. Immediate senior

iii. Head of unit

iv. Head of health facility

From outside the health facility

v. Malaria focal person

vi. Laboratory focal person

vii. Staff from health sub-district

vii. Consultant from the nearest referral hospital

viii. Ministry of Health technical staff

4. How often have you been supervised in the last six months?

i. Once

ii. Twice

iii. Thrice

iv. Monthly

v. None

5. What methods have you been supervised with in the last six months?

i. Direct observation of slides (practical)

ii. Interviews

iii. Inspection

iv. Feedback

v. Problem-solving

vi. Coaching

vii. Training

viii. Decision-making

ix. Clinical audit

x. Other, specify ______________

6. Do you feel support supervision for malaria is useful? ( Y / N )

7. If yes, how is it useful?

i. Improved competence / skills

ii. Improved compliance with national guidelines

iii. Improved effectiveness of care

iv. Improved motivation

v. Other, specify _____________________________________


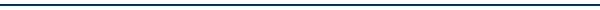


**E. Aides Memoir (AM)**

1. Which of the following malaria laboratory diagnosis aides are **available** at the unit?

i. Posters on the wall ( Y / N )

ii. Wall charts ( Y / N )

ii. Leaflets / Pamphlets ( Y / N )

iii. Reference textbooks ( Y / N )

iv. Desk aids ( Y / N )

Others, specify _________________________________________

2. Which do you prefer as a reminder?

| Rate from 1 to 5 as below |  |
| --- | --- |
| Not useful | 1 |
| A good reminder | 2 |
| A very good reminder | 3 |
| No idea / No response | 4 |
| Not applicable | 5 |

i. Posters on the wall [ ]

ii. Wall charts [ ]

ii. Leaflets / Pamphlets [ ]

iii. Reference textbooks [ ]

iv. Desk aids [ ]

Others, specify _________________________________________

Date: ___ / ____ / 2009 Time _______ am /pm

Completed by: _________________ (name)
